# Asymptomatic Extracranial Artery Stenosis and the Risk of Cardiovascular and Cerebrovascular Diseases

**DOI:** 10.1038/srep33960

**Published:** 2016-09-21

**Authors:** Dandan Wang, Jing Wang, Cheng Jin, Ruijun Ji, Anxin Wang, Xin Li, Xiang Gao, Shouling Wu, Yong Zhou, Xingquan Zhao

**Affiliations:** 1 Department of Neurology, Beijing Tiantan Hospital, Capital Medical University, Beijing, China; 2 China National Clinical Research Center for Neurological Diseases, Beijing, China; 3 Center of Stroke, Beijing Institute for Brain Disorders, Beijing, China; 4 Beijing Key Laboratory of Translational Medicine for Cerebrovascular Disease, Beijing, China; 5 Department of Cardiology, Kailuan Hospital, Tangshan, China; 6 Department of interventional neurology, the affiliated hospital of Qingdao university, Qingdao, China; 7 Department of Nutrition, Harvard University School of Public Health, Boston, MA, USA; 8 Channing Laboratory, Department of Medicine, Brigham and Women’s Hospital, and Harvard Medical School, Boston, MA, USA; 9 Beijing Institute of Heart, Lung and Blood Vessel Diseases, Beijing Anzhen Hospital, Capital Medical University, Beijing, China

## Abstract

Asymptomatic extracranial artery stenosis (ECAS) is a well-known risk factor for stroke events, but it remains unclear whether it has the same role in predicting cardiovascular and cerebrovascular diseases, especially in China. We investigated the potential associations between ECAS, carotid plaque and carotid intima-media thickness and the new occurrence of cardiovascular and cerebrovascular diseases in the study. Out of 5440 study participants, 364 showed an asymptomatic ECAS at baseline, and 185 had come up to the final vascular events (brain infarction, intracerebral hemorrhage, subarachnoid hemorrhage, coronary heart disease and death due to the vascular diseases). During the follow- up. ECAS, carotid plaque and its instability and increased CIMT have associated with vascular events significantly (P < 0.05). After adjusting relevant vascular risk factors, ECAS still has a strong relationship with the new occurrence of vascular events, especially the brain infarction (HR: 2.101; 95% CI: 1.027–4.298; P = 0.042). We observed a clear relationship between ECAS and the new occurrence of cardiovascular and cerebrovascular disease, especially the brain infarction event. Carotid plaque and its instability and increased CIMT have all relevant with the occurrence of vascular events. Our findings provide direct evidence for the importance of ECAS in vascular events occurrence.

Asymptomatic extracranial artery stenosis (ECAS), especially extracranial carotid artery stenosis, is a common disease all around the world, which affects about 7% of women and more than 12% of men in older people^1^. Since ECAS is one of the most important risk factors for ischemic stroke, and other vascular disease and their risk factors such as hypertension and diabetes also have strong relationship with ECAS, the early finding and management of ECAS appears to be essential in preventing and decreasing vascular diseases. However, few studies have examined the impact of asymptomatic ECAS on the risk of cardiovascular and cerebrovascular diseases in Chinese adults. We, therefore, performed a prospective study to examine whether asymptomatic ECAS and related carotid abnormality were associated with a higher risk of cardiovascular and cerebrovascular diseases.

## Results

During the 2-year follow-up, we identified 185 participants who had come up to the final events, which including 59 brain infarction, 12 intracerebral hemorrhage, 3 subarachnoid hemorrhage, 32 coronary heart diseases and 95 deaths. Some participants had occurred more than one outcome event during the follow-up.

Participants who had occur the final events were more likely to be male, older age, had a medical history of hypertension and diabetes, and had a higher level of C-reaction protein, uric acid and homocysteine than the normal participants (Table 1).

There were 364 individuals had ECAS at baseline. We observed significant associations between the occurrence of cardiovascular and cerebrovascular diseases and ECAS, carotid plaque and its instability and thicker CIMT (P < 0.05, Table 2).

To analysis the predictive value of ECAS in different cardiovascular and cerebrovascular diseases, we separated the final events into several sub- groups. We found that ECAS had a higher cumulative incidence of cerebrovascular disease, brain infarction and death, after adjusting relevant vascular risk factors, the significant relationships still almost exist (P < 0.05, Table 3).

## Discussion

In our study population, ECAS has a strong relationship with the new occurrence of cardiovascular and cerebrovascular disease, especially with the brain infarction event. What is more, thicker carotid IMT, carotid plaque and its instability are all relevant with the new occurrence of cardiovascular and cerebrovascular diseases. As we know, this is the first study focus on the predictive value of ECAS to vascular events in Chinese adults.

Previous studies have shown that poor cardiovascular health metrics, including BMI, blood pressure, triglyceride, fast blood glucose, smoking, physical exercise and diet are relevant with the cardiovascular and cerebrovascular disease incidence^2^,^3^. Some common stroke risk factors, such as male, old age, hypertension, diabetes, CRP, uric acid and homocysteine are all relevant with the occurrence of the final events in our study too^4^,^5^.

Cardiovascular and cerebrovascular disease, which both belong to atherothrombotic disease cause a large percentage of death worldwide every year^4^. It will still be the first cause of death by 2020^6^. In Asia, vascular disease is more relevant with cerebrovascular disease than cardiovascular disease^7^. As previous studies indicated that extracranial carotid artery stenosis is a risk factor for stroke event, ECAS is worth to pay more attention to those cerebrovascular patients on their primary and secondary prevention, especially in Asia^1^,^8^,^9^,^10^. In our study, only 3 among the 364 ECAS individuals are extracranial vertebral artery stenosis, rests of them are all extracranial carotid arterial stenosis. So we mainly analysis the relationship between carotid arterial stenosis and final vascular events. Unless cardiovascular disease, ECAS has a strong association with cerebrovascular disease, especially ischemic stroke in our results, even after adjusting all the mentioned vascular risk factors, this relationship still exists significantly. Since cardiovascular and cerebrovascular diseases are not totally same on their pathogenesis, we consider that carotid artery stenosis may influence more on cerebrovascular than cardiovascular, and may cause vascular events more common. Though in the Unite Sates, screening for asymptomatic carotid stenosis is not highly recommended due to its low morbidity to stroke caused by ECAS^1^,^11^, we should not ignore the potential risks of ECAS in Asian people.

CIMT, as well as carotid plaque, are also important indexes on assessing the atherosclerosis level of vascular and predicting the incidence of vascular event. Increased CIMT is a predictive marker for onset of atherosclerosis and is always associated with end-organ disease, while carotid plaque is the marker of early stages of atherosclerosis and advanced arterial injury, which largely reflects the pathological process in the intima^12^,^13^,^14^,^15^. In other study, researchers have demonstrated that increased CIMT and formation of carotid plaque and its instability are relevant of the occurrence of stroke^11^. In our study, we have got the same result. Increased CIMT, formation of carotid plaque and its instability are all associated with the new occurrence of cardiovascular and cerebrovascular disease. So we can conclude that these carotid indexes should also be given full concern on the predictive value of vascular events.

The novelty of our study represented as: First, since the morbidity of carotid atherosclerosis diseases is higher in Europe than in Asia, most of the published papers were based on the European population. There are few studies focused on the asymptomatic carotid atherosclerosis and cardiovascular diseases in Asia, especially in China. But our study proved that ECAS is indeed associated with cardiovascular and cerebrovascular diseases in China. It indicated that Asian people should also focus on the asymptomatic extracranial artery stenosis and vascular events. Secondly, our study is a large community- based study, the population comes from the community instead of hospitals. What is more, we tested the asymptomatic extracranial artery stenosis before the symptoms appears. It makes some help to the first prevention of cardiovascular diseases.

Potential limitations of our study should be discussed. First, the study population was selected only from the participants of the large Kailuan study. They are all the employees and retirees of the Kailuan Company, and most of them are Han Chinese. So some bias may exist because of the population. Secondly, ECAS was evaluated by duplex sonography in our study. The accuracy may vary by different operators. Our study population is an asymptomatic group, and sonography is the most common method on large- scale population screen of carotid artery stenosis worldwide, better and more widely used than Computed Tomography Angiography (CTA), Magnetic Resonance Angiography (MRA) and Digital Subtraction Angiography (DSA) in the cohort population^8^,^9^. Thus, any dissent on the result is discussed and solved by specialist team in our study. Thirdly, severe stenosis is rarely in our population, so we did not analysis the different influence of moderate and severe stenosis on vascular events separately. Fourthly, we did not record which side of the cerebrovascular diseases was effected to the participants during this follow- up period. A larger population and longer- term follow- up are needed to our study in the future.

## Method

### Study Design and Population

The Asymptomatic Polyvascular Abnormalities Community study (APAC) is a community-based, prospective, long-term follow-up observational study, to investigate the epidemiology of asymptomatic polyvascular abnormalities in Chinese adult. The study cohort was a sub-population of a previously described population of the Kailuan study^2^. From June 2010 to June 2011, a sample of 7000 subjects older than 40 years was randomly selected from the Kailuan cohort, and the selected method has been descripted in our previous published protocol^16^. A total of 5440 participants with no history of stroke, transient ischemic attack, and coronary disease at baseline as assessed by a validated questionnaire were included in our final APAC study. The study was performed according to the guidelines from the Helsinki Declaration and was approved by the Ethics Committees of the Kailuan General Hospital and the Beijing Tiantan Hospital. Written informed consent was obtained from all participants.

### Assessment of ECAS

ECAS was evaluated based on a professional duplex sonography (Philips iU-22 ultrasound system, Philips Medical Systems, Bothell, WA). Participant underwent a bilateral carotid sonography at baseline including common carotid arteries, internal carotid artery, external carotid artery, vertebral artery and subclavian artery. An extracranial artery stenosis was defined as an extracranial common or internal carotid artery stenosis, or an extracranial vertebral artery stenosis. The severity of stenosis was graded based on the recommendations from the Society of Radiologists in Ultrasound Consensus Conference, as <50%, 50–69%, >69% and occlusion^17^. If extracranial artery diameter mesurements were not available at baseline, ECAS was defined by a peak systolic blood flow velocity ≥125 cm/s in the common carotid artery or internal carotid artery and ≥170 cm/s in the vertical artery^18^.

### Assesment of other carotid indexes

Carotid intima-media thickness (CIMT) was measured at the far wall of the common carotid artery proximal to the bifurcation, along a plaque-free segment of ≥10 mm long at each side, with a quality index of ≥0.60. CIMTs of bilateral common carotid artery were then averaged to get a mean CIMT value for each subject. The same technician measured CIMTs twice for subsequent assessment of interpreter reproducibility^14^. Carotid plaques were evaluated by plaque complexity and advancement. It was defined as a focal structure either encroaching into the arterial lumen of at least 0.5 mm or 50% of the surrounding IMT value, or demonstrating as a thickness of 1.5 mm from the intima-lumen interface to the media adventitia interface. Unstable plaques in our study were defined as: (1) plaques with incomplete fibrous cap or ulcerated plaques, according to the plaque morphology, and (2) plaques with low-level or heterogeneous echoes, according to the plaque echodensity^19^. The results were reviewed by two independent operators. Discrepancies between their evaluations were resolved by consensus.

### Assessment of epidemiological information and vascular related risk factors

Every participant was taken a standardized questionnaire (age, gender, medical history and other basic information) by our trained investigators. Smoking was defined as at least one cigarette per day for more than a year. Alcohol consumption was defined as an intake of at least 80 g of liquor a day for more than 1 year. Smoking or drinking cessation was considered only if it lasted for at least 1 year. Body weight (to the nearest 0.1 kg) and body height (to the nearest 0.1 cm) were measured, and the body mass index (BMI) was calculated as body weight (kg) divided by the square of height (m2)^18^.

Hypertension was defined as a self- reported history, taking antihypertensive medication, or a systolic blood pressure ≥140 mm Hg, or a diastolic blood pressure of ≥90 mm Hg at baseline. Diabetes mellitus was defined as a self-reported history, current treatment with insulin or oral hypoglycemic agents, or fasting blood glucose level ≥7.0 mmol/l at baseline. Dyslipidemia was defined as a self-reported history, current use of cholesterol lowering medicine, or a total cholesterol level ≥6.22 mmol/l or triglyceride ≥2.26 mmol/l or low density lipoprotein ≥4.14 mmol/l at baseline^20^.

### Follow- up and Outcome Assessment

A face- to- face interview was taken to this cohort at its first follow- up visit up to December 31, 2013, or up to the occurrence of a final event occurs. Physicians and nurses who took this follow- up are masked to the baseline data. Participant who was not able to participate in this follow- up was checked and registered according to his medical records from hospital and medical insurance.

The primary outcome is the first occurrence of cerebrovascular disease, either the first non-fatal stroke event or death by stroke. A non-fatal stroke is defined as a focal neurological deficit of vascular origin and of sudden onset and which lasts >24 hours. Stroke is diagnosed according to the World Health Organization (WHO) criteria combined with brain computed tomography (CT) or magnetic resonance (MR) confirmation, and classified into three main types: brain infarction, intracerebral hemorrhage, and subarachnoid hemorrhage^21^. The criteria are consistent across all participating hospitals. All stroke records are reviewed by two independent stroke specialists. If the two specialists disagree, the event adjudication committee reviews the case and makes the final decision. All stroke outcomes are checked by the Data Safety Monitoring Board and Arbitration Committee for Clinical Outcome^16^.

The first occurrence of cardiovascular disease is an additional outcome event. Incident cardiovascular disease is defined as the only occurrence of a fatal or non-fatal myocardial infarction (I21) during follow-up in our study^22^.

Another outcome event is death due to all the known vascular disease. Participants who died duo to unknown reasons are also included in our outcome event.

### Data Management and Statistical Analyses

The data management system is the SAS software (version 9.3; SAS Institute, Cary, North Carolina, USA). The chi-squared test was used for comparison of categorical variables and t-test was used for continuous variables. Cox proportional hazards regression was used to estimate the events risk by calculating the hazard ratios and 95% confidence intervals. Other relevant risk factors were adjusted during the regression analysis. Total survival rates were shown by different extracranial artery stenosis statuses. The null hypothesis was rejected for P < 0.05.

## Conclusions

We observed a clear relationship between ECAS and the new occurrence of cardiovascular and cerebrovascular disease, especially the brain infarction event. Carotid plaque and its instability and increased CIMT have all relevant with the occurrence of vascular events. Our findings provide direct evidence for the importance of ECAS in vascular events occurrence.

## Additional Information

**How to cite this article**: Wang, D. *et al*. Asymptomatic Extracranial Artery Stenosis and the Risk of Cardiovascular and Cerebrovascular Diseases. *Sci. Rep.* **6**, 33960; doi: 10.1038/srep33960 (2016).

## Figures and Tables

**Table 1:** Baseline characteristics of patients in the Asymptomatic Polyvascular Abnormalities in Community Study.

	no event (n = 5255)	event (n = 185)	p
Male (%)	3122(59.41)	135(72.97)	<0.001
Age (years)	56 ± 12	66 ± 13	<0.001
BMI	24.94 ± 3.25	25.02 ± 3.69	0.732
Smoking	1673(31.84)	64(34.59)	0.429
Alcohol consumption	1739(33.09)	60(32.43)	0.851
hypertension	2482(47.23)	122(65.95)	<0.001
Diabetes	609(11.59)	46(24.86)	<0.001
Dyslipidemia	1625(30.92)	69(37.30)	0.066
CRP*	0.15 ± 1.06	0.68 ± 1.19	<0.001
Uric acid	287.86 ± 89.41	326.63 ± 89.86	<0.001
Hcy*	2.59 ± 0.62	2.83 ± 0.53	<0.001

^*^Recalculated by logarithm events: brain infarction, intracerebral hemorrhage, subarachnoid hemorrhage, myocardial infarction and death

**Table 2:** Crude hazard ratios of ECAS, carotid plaque and its stability and CIMT for events in the Asymptomatic Polyvascular Abnormalities in Community Study (univariate analysis).

	no event	event	p
ECAS	333(6.34)	31(16.76)	<0.001
Carotid plaque	2795(53.19)	149(80.54)	<0.001
Carotid plaque unstable	1438(51.45)	94(63.09)	0.006
Carotid IMT	0.81 ± 0.16	0.92 ± 0.17	<0.001

Events: brain infarction, intracerebral hemorrhage, subarachnoid hemorrhage, myocardial infarction and death.

**Table 3:** Adjusted hazard ratios of ECAS for the presence of events in the Asymptomatic Polyvascular Abnormalities in Community study.

	no ECAS (n = 5076)	ECAS (n = 364)	Model	HR (95%CI)	p
event	154(3.03)	31(8.52)	Model1	3.055(2.077–4.494)	<0.001
			Model2	1.889(1.268–2.814)	0.002
			Model3	1.866(1.252–2.783)	0.002
			Model4	1.785(1.176–2.709)	0.006
cardiovascular disease	29(0.57)	3(0.82)	Model1	1.496(0.456–4.914)	0.506
			Model2	0.947(0.284–3.163)	0.930
			Model3	0.965(0.288–3.233)	0.954
			Model4	0.996(0.295–3.359)	0.995
cerebrovascular disease	60(1.18)	11(3.02)	Model1	2.728(1.434–5.191)	0.002
			Model2	1.972(1.017–3.825)	0.045
			Model3	1.970(1.014–3.829)	0.045
			Model4	1.829(0.908–3.683)	0.091
brain infarction	48(0.95)	11(3.02)	Model1	3.377(1.753–6.503)	<0.001
			Model2	2.293(1.163–4.520)	0.017
			Model3	2.305(1.167–4.553)	0.016
			Model4	2.101(1.027–4.298)	0.042
death	77(1.52)	18(4.95)	Model1	3.445(2.062–5.756)	<0.001
			Model2	1.839(1.076–3.143)	0.026
			Model3	1.790(1.046–3.063)	0.034
			Model4	1.662(0.948–2.915)	0.076
intracerebral hemorrhage	12(0.24)	0(0.00)			
subarachnoid hemorrhage	3(0.06)	0(0.00)			

Event: brain infarction, intracerebral hemorrhage, subarachnoid hemorrhage, myocardial infarction and death cardiovascular disease: myocardial infarction cerebrovascular disease: brain infarction, intracerebral hemorrhage, subarachnoid hemorrhage.

Model 1: unadjusted.

Model 2: adjusted by age, gender.

Model 3: adjusted by age, gender, hypertension, diabetes, hyperlipidemia.

Model 4: adjusted by age, gender, hypertension, diabetes, hyperlipidemia, BMI, CRP, UA, HCY.
